# Identification of a ferroptosis-related gene pair biomarker with immune infiltration landscapes in ischemic stroke: a bioinformatics-based comprehensive study

**DOI:** 10.1186/s12864-022-08295-0

**Published:** 2022-01-16

**Authors:** Jiaxin Fan, Mengying Chen, Shuai Cao, Qingling Yao, Xiaodong Zhang, Shuang Du, Huiyang Qu, Yuxuan Cheng, Shuyin Ma, Meijuan Zhang, Yizhou Huang, Nan Zhang, Kaili Shi, Shuqin Zhan

**Affiliations:** 1 Department of Neurology, The Second Affiliated Hospital of Xi’an Jiaotong University, No. 157 West Five Road, Xi’an, 710004 China; 2 Department of Orthopedics, The Second Affiliated Hospital of Xi’an Jiaotong University, No. 157 West Five Road, Xi’an, 710004 China

**Keywords:** Ischemic stroke, Ferroptosis, Gene pair, Immune infiltration, Biomarker

## Abstract

**Background:**

Ischemic stroke (IS) is a principal contributor to long-term disability in adults. A new cell death mediated by iron is ferroptosis, characterized by lethal aggregation of lipid peroxidation. However, a paucity of ferroptosis-related biomarkers early identify IS until now. This study investigated potential ferroptosis-related gene pair biomarkers in IS and explored their roles in immune infiltration.

**Results:**

In total, we identified 6 differentially expressed ferroptosis-related genes (DEFRGs) in the metadata cohort. Of these genes, 4 DEFRGs were incorporated into the competitive endogenous RNA (ceRNA) network, including 78 lncRNA-miRNA and 16 miRNA-mRNA interactions. Based on relative expression values of DEFRGs, we constructed gene pairs. An integrated scheme consisting of machine learning algorithms, ceRNA network, and gene pair was proposed to screen the key DEFRG biomarkers. The receiver operating characteristic (ROC) curve witnessed that the diagnostic performance of DEFRG pair *CDKN1A/JUN* was superior to that of single gene. Moreover, the CIBERSORT algorithm exhibited immune infiltration landscapes: plasma cells, resting NK cells, and resting mast cells infiltrated less in IS samples than controls. Spearman correlation analysis confirmed a significant correlation between plasma cells and *CDKN1A/JUN* (*CDKN1A*: *r* = − 0.503, *P* < 0.001, *JUN*: *r* = − 0.330, *P* = 0.025).

**Conclusions:**

Our findings suggested that *CDKN1A*/*JUN* could be a robust and promising gene-pair diagnostic biomarker for IS, regulating ferroptosis during IS progression via C9orf106/C9orf139-miR-22-3p-*CDKN1A* and GAS5-miR-139-5p/miR-429-*JUN* axes. Meanwhile, plasma cells might exert a vital interplay in IS immune microenvironment, providing an innovative insight for IS therapeutic target.

**Supplementary Information:**

The online version contains supplementary material available at 10.1186/s12864-022-08295-0.

## Background

According to the latest global burden of neurological disorders statistics, stroke ranks second in the leading cause of death and is the major contributor to long-term disability in adults [1]. Ischemic stroke (IS), making up more than 80% of stroke cases [2], causes by a sudden cessation of local blood flow in a supplying artery to the brain. Following IS attack, ischemic brain tissue suffers from a series of harmful cascade events, including the accumulation of reactive oxygen species, infiltration of immune cells, breakdown of the blood-brain barrier (BBB) as well as irreversible necrosis of neurons [3]. Despite recombinant tissue plasminogen activator (rtPA) has been a mainstay to salvage the ischemic tissue, only a few IS victims can actually benefit from it due to the limitations of narrow therapeutic window, high economic expenditure, and hemorrhage-related complications [4]. Thus, uncovering the potential molecular mechanism and exploring the innovative therapeutic target for IS have been top priorities.

Ferroptosis is a newly recognized type of regulated cell death involved with the intracellular iron-mediated toxic accumulation of lipid peroxidation [5]. Unlike the mitochondrial morphology of other types of cell death, the shrunken mitochondria, increased membrane densities, reduced or disappeared mitochondria crista along with intact nucleus can be observed in ferroptosis under transmission electron microscopy [6–8]. The possible molecular mechanisms of ferroptosis involve abnormal iron metabolism, lipid peroxidation, and some critical enzymes (like GPX4) [9]. Although ferroptosis is first formally put forward in tumors [10], a growing body of work has demonstrated that it is also related to ischemic events, such as in intestinal, lung, and renal [11–13]. In neurology, ferroptosis has been confirmed to participate in intracerebral hemorrhage, subarachnoid hemorrhage, Alzheimer’s disease, amyotrophic lateral sclerosis, and Parkinson’s disease [14–18]. Preliminary evidence reveals that ferroptosis deteriorates ischemia-induced brain damage, while the administration of ferrostatin-1 (an inhibitor of ferroptosis) can effectively reverse induced damage [19, 20]. Recently, increasing studies have focused on finding biomarkers of ferroptosis in multiple levels, including morphology, biochemistry, protein, and gene [21]. For example, Guozhong Chen et al. [22] reported that *MAP1LC3B*, *PTGS2*, and *TLR4* could be potential ferroptosis-related biomarkers for IS via bioinformatics, and further explored potential therapeutic compounds, such as Zinc11679756 (Eltrombopag). However, exploring the reliable predictive gene pair biomarkers and specific regulatory details pertaining to ferroptosis in IS still be enormous challenges.

The ENCODE project changes the perception of noncoding RNAs from junks to essential regulators of cellular homeostasis and disruption [23, 24]. In eukaryotes, noncoding RNAs exert their biological effects in the forms of small (transcripts < 200 nucleotides) RNAs (eg, microRNAs [miRNAs]) and long (transcripts > 200 nucleotides) RNAs (eg, long noncoding RNAs [lncRNAs]) [25]. With the continuous improvement and maturity of RNA sequencing technology and bioinformatics method, a bulk of lncRNAs have been demonstrated to cross-regulate the stability of mRNA at the post-transcriptional level by serving as competing endogenous RNAs (ceRNAs) for shared miRNAs [26]. It is reported that ischemic nerve cells appear more sensitive to the aberrant expression of noncoding RNAs, which affect apoptosis, inflammation, proliferation, autophagy, and angiogenesis [27]. Therefore, further in-depth understanding of these genes may provide a novel perspective for identifiable biomarkers and therapeutic frontier in IS.

As we know, multiple immune cells infiltrate into the ischemic parenchyma from peripheral circulation via broken BBB after IS, triggering innate and adaptive immune responses [28]. Actually, the exact functional roles of activated and infiltrated immune cells depend on the ischemic microenvironment at different phases of IS. For example, neutrophils, dendritic cells, and monocytes appear in the stroked brain, exacerbating neuroinflammatory response by releasing complements, cytokines, cytolysis, as well as interacting with other cells at an early stage after IS. T and B cells infiltrate into the injured brain at chronic stages of IS, facilitating neuron repair and prompting functional recovery [29]. Up to now, few studies have used the CIBERSORT tool to analyze immune infiltration in IS. Hence, evaluating the landscapes of immune infiltration during IS process is of vital significance for advanced targeted therapeutics.

In this study, we firstly identified the differently expressed ferroptosis-related genes (DEFRGs) between IS and control samples from the metadata cohort and ferroptosis-related dataset. According to four independent databases, a ceRNA network was constructed. Then, we used relative expression values of DEFRGs to establish gene pairs. After integrated analysis among least absolute shrinkage and selection operator (LASSO) regression, support vector machine (SVM), ceRNA, and gene pair, we incorporated the key gene-pair biomarker to plot the receiver operating characteristic (ROC) curve and compared their diagnostic capability for IS between gene pair and single gene. Importantly, we further explored the potential regulatory mechanisms of this new biomarker from the perspectives of ceRNA and immune infiltration in IS.

## Methods

### Dataset acquisition and data preprocessing

**Fig. 1:**
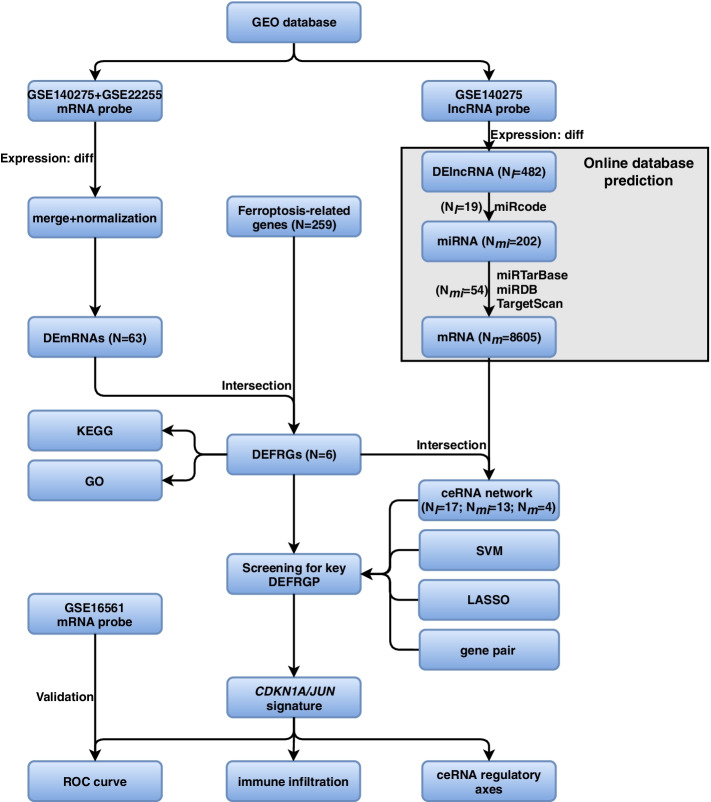
The workflow and data preprocessing of the overall study. (DEmRNAs, differentially expressed mRNAs; DElncRNAs, differentially expressed lncRNAs; DEFRGs, differentially expressed ferroptosis-related genes; DEFRGP, differentially expressed ferroptosis-related gene pair)

We searched the Gene Expression Omnibus (GEO) database (https://www.ncbi.nlm.nih.gov/geo) on 10 September 2020 using the following retrieval conditions: “ischemic stroke” AND “*Homo sapiens*” AND “gse” AND “Expression profiling by array”. We included the gene expression profiling of whole blood or peripheral blood of IS patients or control samples. Profiles with incomplete data, related to cell lines, and associated with other diseases were excluded. Then, two mRNA-sequence datasets (GSE22255 and GSE16561) of 103 patients were retrieved and collected for analysis. Besides, we also downloaded the GSE140275 profile for construction of ceRNA network, which included lncRNA and mRNA expression values of IS. GSE140275 consisted of 3 control and 3 IS samples [30], while GSE22255 was made up of 20 control and 20 IS samples [31]. GSE140275 and GSE22255 datasets served as a discovery cohort. GSE16561 included 24 control and 39 IS samples [32–34], serving as a validation cohort. More detailed information about the three datasets was shown in Table 1. Additionally, a 259 ferroptosis-related genes dataset was fetched from FerrDb [35], including 108 drivers, 69 suppressors, and 111 markers (29 were overlapped genes among them) (More details were shown in Supplemental Table 1). The raw data were preprocessed by the following means: 1) merging GSE140275 and GSE22255 into a metadata cohort to enlarge sample size; 2) carrying out batch normalization to offset the deviations between two datasets using R’s “*sva (v3.34.0)*” package. Because all datasets were publicly accessible from the GEO database or FerrDb database, and the ethics committee approval of the Second Affiliated Hospital of Xi’an Jiaotong University was not required to conduct the current study. Thus, all data were freely available. The workflow and data preprocessing were illustrated in Fig. 1.

**Table 1:** Detailed information of the studied gene expression profiles

Dataset	Platform	Control	IS	Author	Country	Submission	Samples	Application
GSE140275	GPL16791	3	3	Shenghua Li [30]	China	2019	Circulating blood	Identification for DEmRNAs and DElncRNAs
GSE22255	GPL 570	20	20	Sofia A Oliveira [31]	Portugal	2010	Peripheral blood mononuclear cells	Identification for DEmRNAs
GSE16561	GPL6883	24	39	Taura L Barr [32–34]	USA	2009	Peripheral whole blood RNA	Validation for key biomarkers

*IS* Ischemic stroke

### Differential expression analysis

Using R’s “*limma (v3.42.2)*” package to identify differential expressed mRNAs (DEmRNAs) and lncRNAs (DElncRNAs) between IS and control samples. The *P* < 0.05, |log_2_fold change (FC)| > 0.58 or |FC| > 1.5 were selected as the cut-off thresholds, in accordance with previously reported methods [36, 37]. Among DEmRNAs, we took the DEmRNAs that were overlapped with the ferroptosis-related genes as the differentially expressed ferroptosis-related genes (DEFRGs). At the same time, the cluster heatmaps and volcano plots were executed to visualize the difference by R’s “*pheatmap (v1.0.12)*” and “*ggplot2 (v3.3.0)*” packages, respectively.

### Construction of ceRNA network

To explore the potential regulatory mechanisms of DEFRGs, we constructed an intricate ceRNA network. Both lncRNA-miRNA and miRNA-mRNA interactions were obtained through four independent online databases prediction. First, target miRNAs of the above DElncRNAs were predicted by the miRcode database (v11, http://www.mircode.org/). Next, target mRNAs of the obtained miRNAs were predicted by three independent online databases: miRTarBase (v8.0, http://mirtarbase.mbc.nctu.edu.tw/php/index.php), miRDB (v6.0, http://mirdb.org/), and TargetScan (v7.2, http://www.targetscan.org/vert_72/). The upset venn diagram was drawn to search for common predictive target mRNAs shared by any two or three databases. Finally, the overlapped genes between filtrated target mRNAs and DEFRGs were retained as the core of the ceRNA network, which was visualized by Cytoscape software (v3.8.0) [38].

### Functional enrichment analysis

Gene Ontology (GO) terms (consisting of molecular function [MF], biological process [BP], and cellular component [CC]) and Kyoto Encyclopedia of Genes and Genomes (KEGG) pathway (v97.0) [39–41] were used to make a comprehensive investigation for the above DEFRGs based on R’s “*clusterProfiler (v3.14.3)”,* “*enrichplot (v1.6.1)*”, “*org.Hs.eg.db (v3.10.0)*” and “*ggplot2 (v3.3.0)*” packages. The false discovery rate (FDR) adjusted. *P* < 0.05 was set as significant filtering criteria.

### Establishment of gene pair

On the basis of DEFRGs, we established differentially expressed ferroptosis-related gene pairs (DEFRGPs). This method could overcome the technical noise and heterogeneity between different datasets, which has been proven to be effective and reliable [42]. Briefly, each gene pair was calculated by pairwise comparison of the expression value for a given sample. Once the expression value of DEFRG-1 was higher than that of the DEFRG-2 in a specific DEFRGP, the output was defined as 1; otherwise, the output was defined as 0. DEFRG-1 and DEFRG-2 represented any two different DEFRGs. Therefore, 0’s and 1’s formed a ferroptosis-related gene pair. Only the mutual gene pairs in both GSE140275 and GSE22255 were selected as the meaningful DEFRGPs for the subsequent analysis.

### Screening of key DEFRGP biomarker

As a state-of-the-art machine learning algorithm for binary classification, SVM classifies data points by finding a decision boundary to predict labels based on one or more variable vectors [43]. This decision boundary, also called the hyperplane, keeps the margin between classes as far apart as possible [44]. In this study, we addressed the diagnosis prediction of IS as a classification problem (i.e., whether a sample was identified as IS or control). To improve the accuracy of predicting IS outcome, the LASSO regression algorithm was also used to reduce genes dimensionality via seeking for the optimal penalty parameter—λ, which was determined by minimal binomial deviance. To avoid our data suffering from overfitting and find more stable SVM and LASSO regression models, we also performed five-fold cross-validation in these two processes. There were three steps: step 1, divided the data into five equal piles; step 2, selected one pile as testing and the other four piles as training to fit the model; step 3, repeated step 2 five folds in total with different testing selected each time until testing was performed on all five piles. All DEFRGs were subjected to SVM and LASSO regression using R’s “*caret (v6.0-88)*”*,* “*glmnet (v4.1-2)*” packages, respectively. The random seed was set to 3 in all SVM progress, and 214 in all LASSO regression progress. Besides, a venn diagram visualized the key DEFRGP biomarker from the results of LASSO regression, SVM, ceRNA, and gene pair.

### Diagnostic performance of key DEFRGP biomarker in IS

We accessed the diagnostic performance of the key DEFRGP biomarker in the discovery dataset (GSE140275 and GSE22255) using GraphPad Prism software (v8.0.1, GraphPad, Inc., La Jolla, CA, USA). The expression values of gene pair joint diagnosis were obtained via logistic regression. Herein, we compared the discriminative power of single gene and gene pair in terms of the area under the ROC curve (AUCROC), 95% confidence interval (CI), specificity along with sensitivity. Then, we verified the diagnostic performance of key biomarker to distinguish patients with IS from controls in the external validation cohort (GSE16561).

### Immune infiltration analyses

Meanwhile, we quantified the relative percentages of immune cells in each sample using mRNA expression values by CIBERSORT, a classic deconvolution approach based on linear support vector regression [45]. Herein, we performed “*CIBERSORT* (http://cibersort.stanford.edu, accessed on 03 February 2016)” and “*parallel*”, “*e1071 (v1.7-8)*”, “*preprocessCore (v1.48.0)*” packages in R to analyze. The relative percentages of 22 immune cell subpopulations in each individual were visualized by a bar plot. R’s “*corrplot (v0.90)*” package was applied to visualize the association of all cell subpopulations in the form of a correlation heatmap, while the “*ggplot2 (v3.3.0)*” package was utilized to reflect the infiltrating difference between IS and control samples via violin diagram. *P* < 0.05 was accepted as a cut-off value.

### Correlation analysis between immune cell subpopulations and key DEFRGP biomarker

The relationship between the key diagnostic biomarker and immune cell subpopulations in IS was evaluated by Spearman correlation analysis using R’s “*ggstatsplot (v0.9.0)*”, “*limma (v3.42.2)*” packages. And R’s “*ggsci (v2.9)*”, “*ggplot2 (v3.3.0)*”, and “*tidyverse (v1.3.1)*” packages were conducted to visualize the results. *P* < 0.05 was considered significant.

### Statistical analyses

All statistical analyses and graphical work were processed by R software (version 3.6.2, Vienna, Austria). Venn diagrams were conducted using an online tool (http://bioinformatics.psb.ugent.be/webtools/Venn/). The ROC analysis was visualized using GraphPad Prism software (v8.0.1, GraphPad, Inc., La Jolla, CA, USA). Continuous variables were expressed as mean ± SD, and differences between two groups were compared using Student’s t-test for normally distributed variables and Mann–Whitney U test for abnormally distributed variables. Differential expression analysis was performed with the cut-off thresholds of *P* < 0.05 and |log_2_FC| > 0.58 or |FC| > 1.5, which was consistent with previously reported methods [36, 37]. For each study, *P* < 0.05 was considered as a significant difference.

## Results

### Identification of 6 DEFRGs

**Fig. 2:**
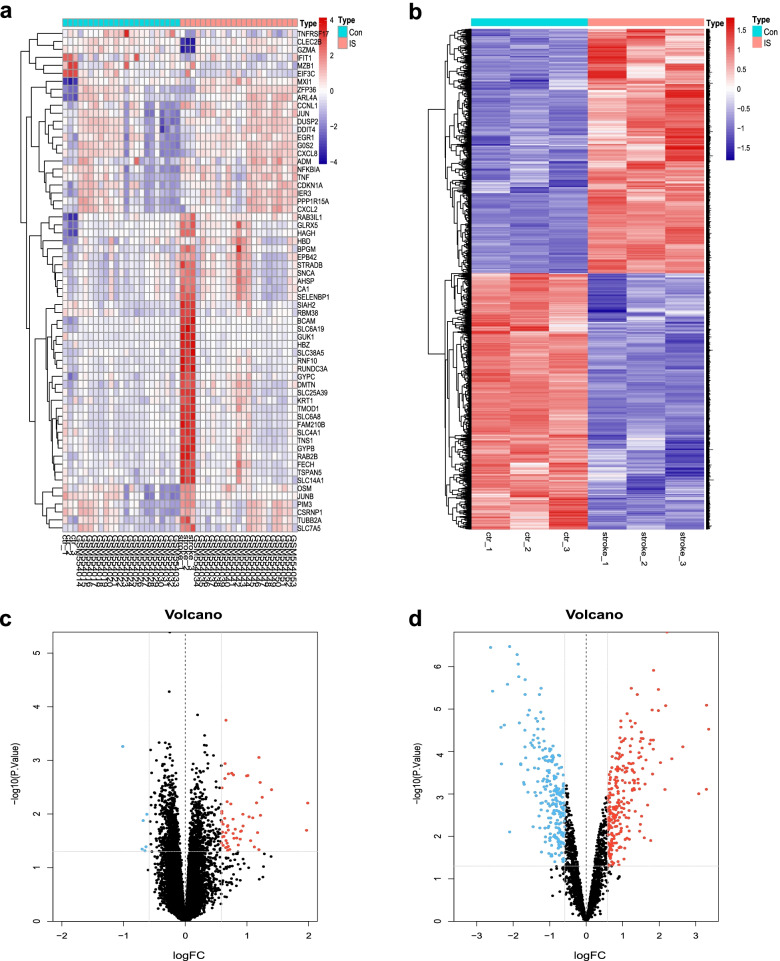
Differential expression analysis. **a** Cluster heatmap for DEmRNAs in metadata cohort. **b** Cluster heatmap for DElncRNAs in GSE140275 dataset. **c** Volcano plot for DEmRNAs in metadata cohort. **d** Volcano plot for DElncRNAs in GSE140275 dataset. Dots in red and blue represent up-regulated and down-regulated differentially expressed genes between ischemic stroke and control samples, respectively. Dots in black represent no differentially expressed genes

A total of 46 patients (23 control and 23 IS samples) were included in this study. When *P* < 0.05 and |log2FC| > 0.58 were used as cut-off thresholds, 63 DEmRNAs in the metadata cohort and 482 DElncRNAs in GSE140275 were directly identified (Fig. 2a, b). The volcano plot illustrated there were 91% (*n* = 58) up-regulated and 9% (*n* = 6) down-regulated genes among DEmRNAs (Fig. 2c). And 51% (*n* = 245) up-regulated and 49% (*n* = 237) down-regulated genes among DElncRNAs (Fig. 2d). After interacting with ferroptosis-related genes, we focused on 6 consistent DEFRGs (*CDKN1A*, *CXCL2*, *DDIT4*, *JUN*, *SLC7A5*, and *ZFP36*) for further analysis, and all of them were up-regulated in IS. More information about them was shown in Table 2.

**Table 2:** Information on the 6 differentially expressed ferroptosis-related genes

Gene	Full name	Protein coded	Role	logFC	***P*** value
CDKN1A	Cyclin dependent kinase inhibitor 1A	Cyclin-dependent kinase inhibitor 1	Suppressor	0.679374	0.006
CXCL2	C-X-C motif chemokine ligand 2	C-X-C motif chemokine 2	Marker	0.860480	0.038
DDIT4	DNA damage inducible transcript 4	DNA damage-inducible transcript 4 protein	Marker	0.586529	0.035
JUN	Jun proto-oncogene, AP-1 transcription factor subunit	Transcription factor AP-1	Suppressor	1.194526	< 0.001
SLC7A5	Solute carrier family 7 member 5	Large neutral amino acids transporter small subunit 1	Marker	0.789610	0.028
ZFP36	ZFP36 ring finger protein	mRNA decay activator protein ZFP36	Suppressor	0.810834	0.019

*FC* Fold change

### Functional enrichment analysis of 6 DEFRGs

**Fig. 3:**
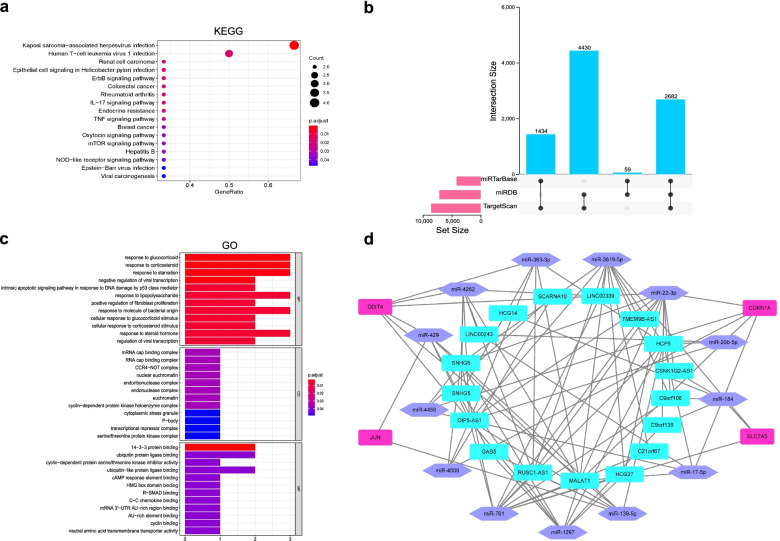
Functional enrichment results and ceRNA network for DEFRGs. **a** Significant enriched KEGG pathways for 6 DEFRGs. **b** Upset venn diagram for filtrating target mRNAs of miRNAs. Three online databases: miRTarBase, miRDB and TargetScan are used to predict target mRNAs of miRNAs, and the screening condition of target mRNAs is that they must be interacted by any two or three databases. **c** Significant enriched GO terms for 6 DEFRGs. (CC, cellular component; BP, biological process; MF, molecular function). **d** A ceRNA network is constructed via 17 lncRNAs (cyanine rectangle), their 13 corresponding miRNAs (purple hexagon) as well as 4 DEFRGs (purple pink rectangle), and lines represented their interactions

KEGG enrichment uncovered the above 6 DEFRGs principally participated in renal cell carcinoma, colorectal cancer, rheumatoid arthritis, breast cancer, IL-17 signaling pathway, endocrine resistance, TNF signaling pathway, oxytocin signaling pathway, mTOR signaling pathway, hepatitis B, NOD-like receptor signaling pathway (Fig. 3a and Supplemental Table 2). BP was mainly enriched in the response to glucocorticoid, response to starvation, intrinsic apoptotic signaling pathway in response to DNA damage by p53 class mediator, response to lipopolysaccharide, positive regulation of fibroblast proliferation, and response to steroid hormone. CC showed that 6 DEFRGs were related to nuclear euchromatin, euchromatin, cyclin-dependent protein kinase holoenzyme complex, transcriptional repressor complex, and serine/threonine protein kinase complex. In MF, 6 DEFRGs were mainly associated with ubiquitin protein ligase binding, cAMP response element binding, HMG box domain binding, R-SMAD binding, cyclin binding, and neutral amino acid transmembrane transporter activity (Fig. 3c and Supplemental Table 3).

### Construction of ceRNA network

The miRcode database prediction displayed 19 DElncRNAs had binding sites with 202 miRNAs among identified 482 DElncRNAs. And the combined prediction of miRDB, miRTarBase, and TargetScan databases demonstrated that 54 obtained miRNAs could bind to 8605 target mRNAs among the aforesaid 202 miRNAs (Figs. 1 and 3b). We merged these 8605 predictive mRNAs with 6 DEFRGs, retaining 4 overlapped DEFRGs (*CDKN1A*, *DDIT4*, *JUN*, and *SLC7A5*) as the core of the ceRNA network. Eventually, a ceRNA network was constructed via 34 nodes and 94 edges. Specifically, there were 17 lncRNA nodes, their 13 corresponding miRNA nodes as well as 4 DEFRG nodes. The 94 edges represented 78 lncRNA-miRNA and 16 miRNA-mRNA interactions (Fig. 3d).

### Screening for key DEFRGP biomarker

**Fig. 4:**
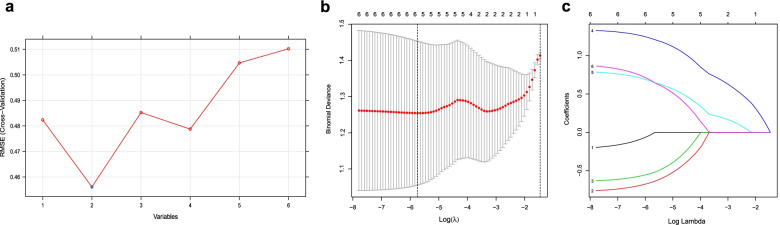
Screening for key DEFRGs using machine learning algorithms. **a** Screening for key DEFRGs by SVM. **b**, **c** Screening for key DEFRGs by LASSO regression. **b** Binomial deviance profiles of DEFRGs in metadata cohort. **c** The LASSO coefficient is plotted against log lambda in metadata cohort

**Fig. 5:**
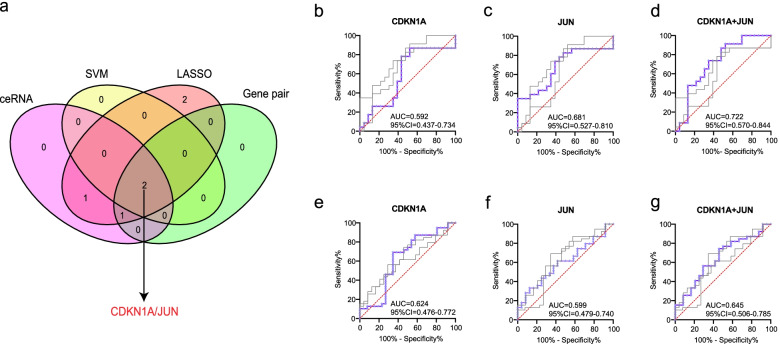
Integration scheme and diagnostic performance of *CDKN1A/JUN* in predicting ischemic stroke. **a** A 4 set venn diagram shows the integration strategy among ceRNA, SVM, LASSO regression, and gene pair. The purple pink circle represents for DEFRGs of ceRNA network, the yellow circle represents for DEFRGs screened by SVM, the peach pink circle represents for DEFRGs screened by LASSO regression, the green circle represents for meaningful DEFRGPs in both GSE140275 and GSE22255. As shown, *CDKN1A/JUN* is the key gene pair. **b**-**d** Comparison of ROC analysis between gene pair and single gene in the discovery cohort. **b** ROCs for *CDKN1A*. **c** ROCs for *JUN*. **d** ROCs for *CDKN1A*/*JUN* gene pair. **e**-**g** Comparison of ROC analysis between gene pair and single gene in the validation cohort. **e** ROCs for *CDKN1A*. **f** ROCs for *JUN*. **g** ROCs for *CDKN1A*/*JUN* gene pair

In the metadata cohort, we totally established 2 meaningful DEFRGPs: *CDKN1A*/*JUN*, *JUN*/*SLC7A5*. SVM created a hyperplane for 2 DEFRGs (*CDKN1A*, *JUN*) at the seed of 3 (Fig. 4a). While LASSO regression obtained the minimum binomial deviance at the seed of 214 (Fig. 4b, c), keeping 6 DEFRGs perfectly. The venn diagram displayed that *CDKN1A*/*JUN* was screened as the key DEFRGP biomarker among the results of LASSO regression, SVM, gene pair, and ceRNA (Fig. 5a).

### Diagnostic performance of *CDKN1A/JUN* in IS

The diagnostic performances of the key gene pair (*CDKN1A*/*JUN*) and genes (*CDKN1A* and *JUN*) to distinguish patients with IS and controls were appraised via ROC analysis in the discovery and validation cohorts. The AUCROC was 0.592 (95%CI = 0.437-0.734, sensitivity = 86.96%, specificity = 47.83%) for *CDKN1A*, 0.681 (95%CI = 0.527-0.810, sensitivity = 82.61%, specificity = 52.17%) for *JUN*, and 0.722 (95%CI = 0.570-0.844, sensitivity = 91.30%, specificity = 47.83%) when these two genes combined into gene pair in the GSE140275 and GSE22255 datasets (Fig. 5b-d). And the AUCROC was 0.624 (95%CI = 0.476-0.772, sensitivity = 69.23%, specificity = 65.38%) for *CDKN1A*, 0.599 (95%CI = 0.479-0.740, sensitivity = 33.33%, specificity = 87.5%) for *JUN*, and 0.645 (95%CI = 0.506-0.785, sensitivity = 74.36%, specificity = 54.17%) when these two genes combined into gene pair in the GSE16561 dataset (Fig. 5e-g). Compared to *CDKN1A* and *JUN*, the *CDKN1A*/*JUN* gene pair exhibited superior discriminative effectiveness in predicting IS. And the results for the external validation dataset also witnessed the effectiveness and robustness of *CDKN1A*/*JUN* gene pair.

### Immune infiltration landscapes

**Fig. 6:**
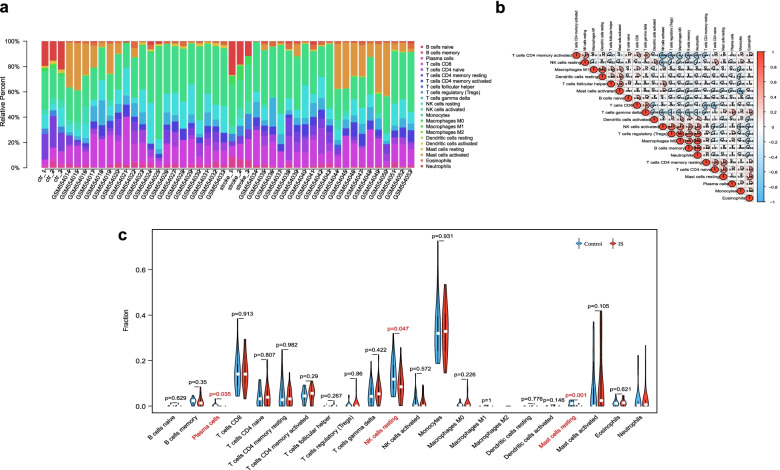
Immune infiltration landscapes in metadata cohort. **a** Bar plot shows the relative percent of 22 immune cell subpopulations in each individual. **b** Correlation heatmap of 22 immune cell subpopulations. Red and blue represent positive and negative correlation, respectively. The circle with a deeper color and larger fill area has a stronger correlation index. **c** Violin diagram displays different fractions of 22 immune cell subpopulations in IS and control samples. The red marks represent significant infiltrating difference of subpopulations

To understand the roles of *CDKN1A/JUN* in the brain microenvironment during IS process, we investigated immune cells landscapes and their relationship with *CDKN1A/JUN*. The bar plot clearly showed that the contents of varied subpopulations in each individual (Fig. 6a). Correlation heatmap between 22 immune cell subpopulations in IS revealed that M1 macrophages were positively correlated with resting dendritic cells. Regulatory T cells displayed distinct associations with M0 macrophages, memory B cells, and neutrophils. M0 macrophages were positively correlated with memory B cells and neutrophils, and the latter two also showed a significantly positive correlation (Fig. 6b). The violin diagram showed that compared with control samples, plasma cells, resting NK cells, and resting mast cells were all presented with lower infiltrates in IS samples (Fig. 6c).

**Fig. 7:**
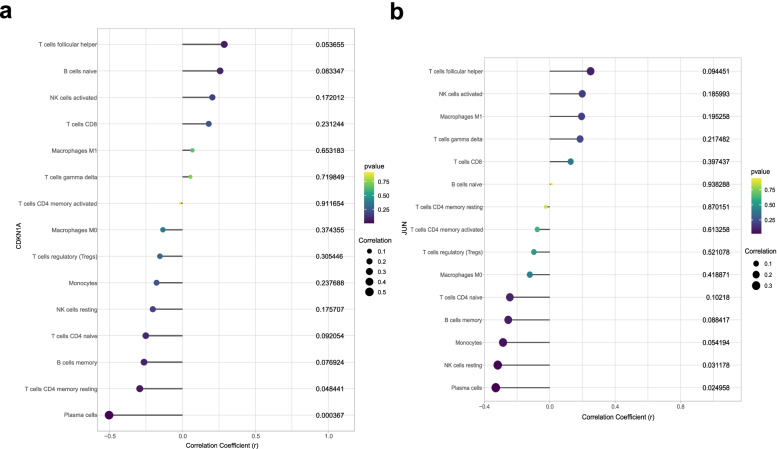
Visualization of Spearman correlation between immune cell subpopulations and *CDKN1A/JUN*. **a** Correlation analysis between immune cell subpopulations and *CDKN1A*. **b** Correlation analysis between immune subpopulations and *JUN*. The dot with a larger size has a stronger correlation coefficient. The *p* value is presented by different color, the dot with a more purple color has a smaller *p* value, while the dot with a yellower color has a larger *p* value

Intriguingly, we found a substantial relationship between *CDKN1A/JUN* and plasma cells among the three infiltrating cell subpopulations. Specifically, *CDKN1A* had a negative correlation with plasma cells (*r* = − 0.503, *P* < 0.001) (Fig. 7a and Supplemental Table 4), while *JUN* was not only negatively correlated with plasma cells (*r* = − 0.330, *P* = 0.025) but also with resting NK cells (*r* = − 0.318, *P* = 0.031) (Fig. 7b and Supplemental Table 5). However, *CDKN1A* had no correlation with resting NK cells (Fig. 7a). Besides, the resting mast cell was not associated with either *CDKN1A* or *JUN*. These results suggested that *CDKN1A*/*JUN* could partly reflect the condition of the brain microenvironment in IS.

### Exploration of potential regulatory axes for *CDKN1A/JUN*

**Fig. 8:**
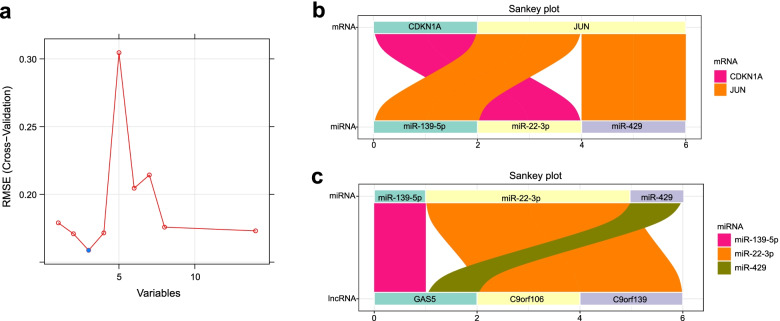
Refining for core lncRNA using SVM and ceRNA regulatory axes for *CDKN1A/JUN*. **a** Refining for core lncRNA by SVM. **b-c** The ceRNA regulatory axes for *CDKN1A/JUN* biomarker. **b** Sankey plot displays the interacted miRNAs for *CDKN1A* and *JUN*. **c** Sankey plot displays the interacted lncRNAs for the above miRNA

After screening out the robust *CDKN1A/JUN* biomarker to identifying patients with IS, we further explored their regulatory axes according to the ceRNA network. Given that a miRNA could bind to multiple lncRNAs and 14 lncRNAs were too many to accurately mine the potential ceRNA axes for *CDKN1A/JUN* biomarker. Accordingly, SVM was utilized again to refine the upstream regulated lncRNAs, refining 3 core lncRNAs (C9orf106, C9orf139, GAS5) at the seed of 9999 (Fig. 8a). In terms of *CDKN1A*, only miR-22-3p had a binding site for it, while C9orf106 and C9orf139 could co-regulate miR-22-3p; for *JUN*, miR-139-5p and miR-429 could co-bind to it, while both of them were regulated by GAS5 (Fig. 8b, c).

## Discussion

Despite the great improvement that has been made in IS treatment over decades, the thrombolytic therapy is nevertheless not satisfactory, and a number of patients still suffer from long-term disabilities. Along with the development of sequencing technology, an expectation increasingly grows that exploring the biological effects of noncoding RNAs in the non-oncology field, especially in stroke. Ferroptosis has been confirmed to be involved in the occurrence and development of IS. However, very few studies have focused on ferroptosis-related genes and potential regulatory details as well as the immune infiltration landscapes, which has profound significance for IS patients. In the current study, totally 6 DEFRGs were identified in the metadata cohort. The integration scheme among LASSO regression, SVM, ceRNA, and gene pair revealed that *CDKN1A*/*JUN* was the key ferroptosis-related gene pair. In addition, CIBERSORT was applied to analyze immune infiltration in IS.

Compared to prior studies [46], a merge of datasets and integration scheme were two dominant advantages in this study. On the one hand, merging datasets allowed for a larger sample size to incorporate more DEFRGs, which was conducive to subsequent machine learning analysis. On the other hand, the ROC analysis results showed that the superior discriminative effectiveness of *CDKN1A*/*JUN* gene pair than single gene (*CDKN1A* and *JUN*) both in the discovery and validation cohorts, implying that the integration scheme was feasible and reliable. Cyclin-dependent kinase inhibitor 1A (*CDKN1A*), also known as p21^WAF1/Cip1^, interacts with cyclin-dependent kinases to exert its activity [47]. Accumulating evidence implied that up-regulated expression of *CDKN1A* led to the block of cell cycle at the G1 phase in a *p53*-dependent or *p53*-independent manner, and then induced apoptosis [48–50]. Apart from apoptosis, *CDKN1A* was also reported to induce autophagy via ursolic acid [51]. One previous clinical research indicated that *CDKN1A* participated in the proliferation of mesenchymal stem cells in humans with IS serum [52]. Besides, *CDKN1A* could impact peroxide metabolism, such as glutathione, making it an ideal candidate for detecting ferroptosis [53]. Considering that *CDKN1A* always involved in cell death, it was reasonable to speculate that it played a pivotal role in ferroptosis via regulating the cell cycle process in IS. The c-JUN protein encoded by *JUN,* acting as a transcription factor, dimerizes with Maf/Nrl families or Fos families to regulate gene transcription [54]. In mammals, c-JUN takes part in diverse cell activities and pathophysiologic processes, including proliferation, differentiation, senescence, apoptosis, neuronal development, inflammations, tumorigenesis as well as cellular transformation [55, 56]. Li Y et al. confirmed that the expression of c-JUN was up-regulated, which aggravated cerebral ischemia/reperfusion (I/R) induced injury [57]. As the downstream effector of the JNK pathway, c-JUN was found to regulate inflammation and cell death in the ischemic brain. Moreover, c-JUN was also implicated to regulate cell pyroptosis and activate the NLRP3 inflammasome [58]. Thus, we reckoned that *JUN* played a momentous part in the progression of IS. Known evidence from prior researches together with our findings suggested that the functions and effects of *CDKN1A* and *JUN* in IS should be the center of investigations in the near future.

A plethora of data has indicated the participation of ferroptosis in immunity [5, 59]. Ferroptotic cells can activate innate immunity and release pro-inflammatory factors in various diseases (including myocardial I/R injury, and glioma), recruiting lots of immune cells [60]. When IS occurs, the breakdown of BBB allows immune cells to flood into the central nervous system. For instance, Meng H et al. found that double-negative T cells were gradually increased in a time-dependent manner, amplifying pro-inflammatory microglia and prompting brain injury in IS patients or MCAO mice [61]. Our findings suggested that plasma cells, resting NK cells, and resting mast cells infiltrated less in IS samples compared to controls. Remarkably, only plasma cells were linked to *CDKN1A*/*JUN* biomarker simultaneously. Kong Y et al. [62] reported that the number of NK cells reduced in IS patients, keeping in accordance with our findings. Mast cells played a detrimental role in IS by accelerating BBB disruption and magnifying neuroinflammation via releasing cytokines [63]. Another in vivo model confirmed that the inflammation in infarcts, especially mediated by increased B cells or plasma cells, contributed to post-stroke cognitive impairment by secreting antibodies or complements [64], which was contrary to our findings. One plausible explanation was that our gene expression data came from the serum, not the brain tissue, plasma cells would migrate from circulation to ischemic brain tissue to protect neurons from inflammatory damage after IS. Further research is warranted to address this contradictory issue.

The potential regulatory mechanisms through which *CDKN1A*/*JUN* carried out are also noteworthy. Based on the ceRNA network and screen of core lncRNAs via SVM, our results showed potential regulatory axes for *CDKN1A* and *JUN*. Both C9orf106 and C9orf139 could act as sponges of miR-22-3P, competing bind to up-regulated *CDKN1A*. Through promoting the polarization of macrophages, miR-22-3p inhibited inflammation and lessened the spinal cord I/R injury [65]. Nevertheless, there were few works about C9orf106 and C9orf139. Both miR-139-5p and miR-429 had binding sites for *JUN*, while GAS5 abolished this effect via sponging miR-139-5p or miR-429. Several lines of evidence showed that GAS5 was extensively related to neuronal apoptosis and differentiation [66]. And a previous study implicated that the miR-139-5p/c-JUN-initiated pathway regulated the function of diabetic endothelial cells, providing an experimental basis for our findings [67]. Another evidence revealed that miR-429 inhibited hepatocyte proliferation via negatively regulating c-JUN [68]. Unfortunately, the expression profile for miRNAs was rare in the GEO database. Hence, it is undeniable that more work is needed to explore and confirm our findings.

According to KEGG enrichment analysis, these 6 DEFRGs principally participated in TNF signaling pathway, mTOR signaling pathway, NOD-like receptor signaling pathway. Existing evidence lent support to the idea that these pathways affected the initiation and progression of IS. For example, TNF and IL-1β accelerated inflammatory lesions in IS via NOD-like receptor signaling pathway [69]. GO enrichment analysis revealed that these genes mainly enriched in intrinsic apoptotic signaling pathway in response to DNA damage by p53 class mediator in BP, correlating with cyclin-dependent protein kinase holoenzyme complex. Previous research supported our findings, indicating that DNA damage-signaling pathway responses aggravated brain I/R injury and this process could be attenuated by chloroquine [70]. Cyclin-dependent protein kinase holoenzyme complex was a crucial regulator in the cell cycle involved in many processes, including apoptosis, senescence, and autophagy. Additionally, they were mainly associated with cAMP response element binding in MF, whose phosphorylation was indispensable for the decrease in oxygen–glucose deprivation and reoxygenation-induced apoptosis of astrocytes [71].

Despite we merged two different datasets to enlarge the sample size, there were several limitations that should be acknowledged. First, it was a retrospective analysis, bringing about an inherent bias. Second, the profiles of our datasets were from the blood samples instead of the brain tissue, their reliability should be verified henceforth. Third, the *CDKN1A/JUN* biomarker was constructed on RNA sequences, their reproducibility and wide applicability need to be validated using experimental or clinical samples.

## Conclusions

Taken together, our findings indicated that the *CDKN1A/JUN* was a robust and promising diagnostic biomarker for identifying the patients with IS, which might regulate ferroptosis during the progression of IS via C9orf106/C9orf139-miR-22-3p-*CDKN1A* and GAS5-miR-139-5p/miR-429-*JUN* axes. Meanwhile, plasma cells might exert a vital interplay in the new gene pair biomarker’s diagnostic ability, providing an innovative insight for IS therapeutic target. Further biological experiments in combination with larger prospective clinical samples are warranted to verify the functions and effects of this new gene pair biomarker in IS.

## Supplementary Information

**Additional file 1: Supplemental Table 1.** The 259 ferroptosis-related genes. **Supplemental Table 2.** Significant KEGG pathways for 6 differentially expressed ferroptosis-related genes. **Supplemental Table 3.** Significant GO terms for 6 differentially expressed ferroptosis-related genes. **Supplemental Table 4.** Spearman correlation between immune cell subpopulations and *CDKN1A*. **Supplemental Table 5.** Spearman correlation between immune cell subpopulations and *JUN*.

## Data Availability

The datasets that support the findings of the current study are available in the GEO database (https://www.ncbi.nlm.nih.gov/geo) and FerrDb database. And they are also available from the corresponding author, upon reasonable request. Details about GSE140275, GSE22255, and GSE16561 are shown in Table 1, details pertaining to ferroptosis-related genes are shown in Supplemental Table 1.

## Abbreviations

IS: Ischemic stroke
DEFRG: Differentially expressed ferroptosis-related gene
DEFRGP: Differentially expressed ferroptosis-related gene pair
ceRNA: Competitive endogenous RNA
LASSO: Least absolute shrinkage and selection operator
SVM: Support vector machine
GO: Gene Ontology
KEGG: Kyoto Encyclopedia of Genes and Genomes
MF: Molecular function
BP: Biological process
CC: Cellular component
ROC: Receiver operating characteristic
AUCROC: The area under ROC curve
BBB: Blood-brain barrier
GEO: Gene Expression Omnibus
lncRNAs: Long noncoding RNAs
miRNAs: microRNAs
