# Supplementary material for: Identification of a ferroptosis-related gene pair biomarker with immune infiltration landscapes in ischemic stroke: a bioinformatics-based comprehensive study
Source: BMC Genomics. 2022 Jan 16;23:59. doi: 10.1186/s12864-022-08295-0 (PMC8761271; doi:10.1186/s12864-022-08295-0)
Supplement: Supplementary file 1 — Additional file 1: Supplemental Table 1. The 259 ferroptosis-related genes. Supplemental Table 2. Significant KEGG pathways for 6 differentially expressed ferroptosis-related genes. Supplemental Table 3. Significant GO terms for 6 differentially expressed ferroptosis-related genes. Supplemental Table 4. Spearman correlation between immune cell subpopulations and CDKN1A. Supplemental Table 5. Spearman correlation between immune cell subpopulations and JUN. [file 12864_2022_8295_MOESM1_ESM.docx]

**Supplementary materials**

**Supplemental Table 1.** The 259 ferroptosis-related genes.

| **Genes** | **Full names** | **Protein encoded** | **Genes** | **Full names** | **Protein encoded** |  |
| --- | --- | --- | --- | --- | --- | --- |
| **Drivers** |  |  |  |  |  |  |
| RPL8 | Ribosomal protein L8 | 60S ribosomal protein L8 | IREB2 | Iron response element binding protein 2 | Iron-responsive element-binding protein 2 |  |
| ATP5MC3 | ATP synthase membrane subunit c locus 3 | ATP synthase F(0) complex subunit C3 | CS | Citrate synthase | Citrate synthase |  |
| EMC2 | ER membrane protein complex subunit 2 | ER membrane protein complex subunit 2 | ACSF2 | Acyl-CoA synthetase family member 2 | Medium-chain acyl-CoA ligase ACSF2 |  |
| NOX1 | Nicotinamide adenine dinucleotide phosphate (NADPH) oxidase (NOX) 1 | NADPH oxidase 1 | CYBB | Cytochrome b-245 beta chain | Cytochrome b-245 heavy chain |  |
| NOX3 | Nicotinamide adenine dinucleotide phosphate (NADPH) oxidase (NOX) 3 | NADPH oxidase 3 | NOX4 | Nicotinamide adenine dinucleotide phosphate (NADPH) oxidase (NOX) 4 | NADPH oxidase 4 |  |
| NOX5 | Nicotinamide adenine dinucleotide phosphate (NADPH) oxidase (NOX) 5 | NADPH oxidase 5 | ATG16L1 | Autophagy related 16 like 1 | Autophagy-related protein 16-1 |  |
| DUOX2 | Dual oxidase 2 | Dual oxidase 2 | G6PD | Glucose-6-phosphate dehydrogenase | Glucose-6-phosphate 1-dehydrogenase |  |
| PGD | Phosphoglycerate dehydrogenase | 6-phosphogluconate dehydrogenase | VDAC2 | Valtage-dependent anion channels 2 | Voltage-dependent anion-selective channel protein 2 |  |
| PIK3CA | Phosphatidylinositol-4,5-bisphosphate 3-kinase catalytic subunit alpha | Phosphatidylinositol 4 | FLT3 | Fms related tyrosine kinase 3 | Receptor-type tyrosine-protein kinase FLT3 |  |
| SCP2 | Sterol carrier protein 2 | Non-specific lipid-transfer protein | TP53 | Tumor protein p53 | Cellular tumor antigen p53 |  |
| ACSL4 | Acyl-CoA synthetase long chain family member 4 | Long-chain-fatty-acid--CoA ligase 4 | LPCAT3 | Lysophosphatidylcholine acyltransferase 3 | Lysophospholipid acyltransferase 5 |  |
| NRAS | NRAS proto-oncogene, GTPase | GTPase NRas | KRAS | KRAS proto-oncogene, GTPase | GTPase KRas |  |
| HRAS | HRas proto-oncogene, GTPase | GTPase HRas | TF | Transferrin | Serotransferrin |  |
| TFRC | Transferrin receptor | Transferrin receptor protein 1 | TFR2 | Transferrin receptor 2 | Transferrin receptor protein 2 |  |
| SLC38A1 | Solute carrier family 38 member 1 | Sodium-coupled neutral amino acid transporter 1 | SLC1A5 | Solute carrier family 1 member 5 | Neutral amino acid transporter B(0) |  |
| GABARAPL1 | GABA type A receptor associated protein like 1 | Gamma-aminobutyric acid receptor-associated protein-like 1 | GOT1 | Glutamic-oxaloacetic transaminase 1 | Aspartate aminotransferase |  |
| ALOX5 | Arachidonate 5-lipoxygenase | Arachidonate 5-lipoxygenase | KEAP1 | Kelch like ECH associated protein 1 | Kelch-like ECH-associated protein 1 |  |
| HMOX1 | Heme oxygenase 1 | Heme oxygenase 1 | ATG5 | Autophagy related 5 | Autophagy protein 5 |  |
| ATG7 | Autophagy related 7 | Ubiquitin-like modifier-activating enzyme ATG7 | NCOA4 | Nuclear receptor coactivator 4 | Nuclear receptor coactivator 4 |  |
| ALOX12 | Arachidonate 12-lipoxygenase, 12S type | Arachidonate 12-lipoxygenase | ALOX12B | Arachidonate 12-lipoxygenase, 12R type | Arachidonate 12-lipoxygenase |  |
| ALOX15 | Arachidonate 15-lipoxygenase | Arachidonate 15-lipoxygenase | ALOX15B | Arachidonate 15-lipoxygenase type B | Arachidonate 15-lipoxygenase B |  |
| ALOXE3 | Arachidonate lipoxygenase 3 | Hydroperoxide isomerase ALOXE3 | PHKG2 | Phosphorylase kinase catalytic subunit gamma 2 | Phosphorylase b kinase gamma catalytic chain |  |
| ACO1 | Aconitase 1 | Cytoplasmic aconitate hydratase | G6PDX | _NA_ | _NA_ |  |
| ULK1 | Unc-51 like autophagy activating kinase 1 | Serine/threonine-protein kinase ULK1 | ATG3 | Autophagy related 3 | Ubiquitin-like-conjugating enzyme ATG3 |  |
| GABARAPL2 | GABA type A receptor associated protein like 2 | Gamma-aminobutyric acid receptor-associated protein-like 2 | BECN1 | Beclin 1 | Beclin-1 |  |
| PEBP1 | Phosphatidylethanolamine binding protein 1 | Phosphatidylethanolamine-binding protein 1 | ATG4D | Autophagy related 4D cysteine peptidase | Cysteine protease ATG4D |  |
| GLS2 | Glutaminase 2 | Glutaminase liver isoform | DUOX1 | Dual oxidase 1 | Dual oxidase 1 |  |
| WIPI1 | WD repeat domain, phosphoinositide interacting 1 | WD repeat domain phosphoinositide-interacting protein 1 | WIPI2 | WD repeat domain, phosphoinositide interacting 2 | WD repeat domain phosphoinositide-interacting protein 2 |  |
| SNX4 | Sorting nexin 4 | Sorting nexin-4 | ATG13 | Autophagy related 13 | Autophagy-related protein 13 |  |
| ULK2 | Unc-51 like autophagy activating kinase 2 | Serine/threonine-protein kinase ULK2 | SAT1 | Spermidine/spermine N1-acetyltransferase 1 | Diamine acetyltransferase 1 |  |
| EGFR | Epidermal growth factor receptor | Epidermal growth factor receptor | MAPK3 | Mitogen-activated protein kinase 3 | Mitogen-activated protein kinase 3 |  |
| MAPK1 | Mitogen-activated protein kinase 1 | Mitogen-activated protein kinase 1 | BID | BH3 interacting domain death agonist | BH3-interacting domain death agonist |  |
| ZEB1 | Zinc finger E-box binding homeobox 1 | Zinc finger E-box-binding homeobox 1 | DPP4 | Dipeptidyl peptidase 4 | Dipeptidyl peptidase 4 |  |
| CDKN2A | Cyclin dependent kinase inhibitor 2A | Cyclin-dependent kinase inhibitor 2A | MAP1LC3A | Microtubule associated protein 1 light chain 3 alpha | Microtubule-associated proteins 1A/1B light chain 3A |  |
| SOCS1 | Suppressor of cytokine signaling 1 | Suppressor of cytokine signaling 1 | CDO1 | Cysteine dioxygenase type 1 | Cysteine dioxygenase type 1 |  |
| MYB | MYB proto-oncogene, transcription factor | Transcriptional activator Myb | MAPK8 | Mitogen-activated protein kinase 8 | Mitogen-activated protein kinase 8 |  |
| MAPK9 | Mitogen-activated protein kinase 9 | Mitogen-activated protein kinase 9 | CHAC1 | ChaC glutathione specific gamma-glutamylcyclotransferase 1 | Glutathione-specific gamma-glutamylcyclotransferase 1 |  |
| BACH1 | BTB domain and CNC homolog 1 | Transcription regulator protein BACH1 | HIF1A | Hypoxia inducible factor 1 subunit alpha | Hypoxia-inducible factor 1-alpha |  |
| PRKAA2 | Protein kinase AMP-activated catalytic subunit alpha 2 | 5'-AMP-activated protein kinase catalytic subunit alpha-2 | PRKAA1 | Protein kinase AMP-activated catalytic subunit alpha 1 | 5'-AMP-activated protein kinase catalytic subunit alpha-1 |  |
| ELAVL1 | ELAV like RNA binding protein 1 | ELAV-like protein 1 | BAP1 | BRCA1 associated protein 1 | Ubiquitin carboxyl-terminal hydrolase BAP1 |  |
| ABCC1 | ATP binding cassette subfamily C member 1 | Multidrug resistance-associated protein 1 | MIR6852 | microRNA 6852 | _NA_ |  |
| ACVR1B | Activin A receptor type 1B | Activin receptor type-1B | TGFBR1 | Transforming growth factor beta receptor 1 | TGF-beta receptor type-1 |  |
| EPAS1 | Endothelial PAS domain protein 1 | Endothelial PAS domain-containing protein 1 | HILPDA | Hypoxia inducible lipid droplet associated | Hypoxia-inducible lipid droplet-associated protein |  |
| LINC00472 | Long intergenic non-protein coding RNA 472 | Putative uncharacterized protein encoded by LINC00472 | IFNG | Interferon gamma | Interferon gamma |  |
| ANO6 | Anoctamin 6 | Anoctamin-6 | LPIN1 | Lipin 1 | Phosphatidate phosphatase LPIN1 |  |
| HMGB1 | High mobility group box 1 | High mobility group protein B1 | TNFAIP3 | TNF alpha induced protein 3 | Tumor necrosis factor alpha-induced protein 3 |  |
| TLR4 | Toll like receptor 4 | Toll-like receptor 4 | ATF3 | Activating transcription factor 3 | Cyclic AMP-dependent transcription factor ATF-3 |  |
| ATM | ATM serine/threonine kinase | Serine-protein kinase ATM | YY1AP1 | YY1 associated protein 1 | YY1-associated protein 1 |  |
| EGLN2 | Egl-9 family hypoxia inducible factor 2 | Egl nine homolog 2 | MIOX | Myo-inositol oxygenase | Inositol oxygenase |  |
| TAZ | Tafazzin | Tafazzin | MTDH | Metadherin | Protein LYRIC |  |
| IDH1 | Isocitrate dehydrogenase (NADP(+)) 1 | Isocitrate dehydrogenase [NADP] cytoplasmic | SIRT1 | Sirtuin 1 | NAD-dependent protein deacetylase sirtuin-1 |  |
| FBXW7 | F-box and WD repeat domain containing 7 | F-box/WD repeat-containing protein 7 | PANX1 | Pannexin 1 | Pannexin-1 |  |
| DNAJB6 | DnaJ heat shock protein family (Hsp40) member B6 | DnaJ homolog subfamily B member 6 | MAPK14 | Mitogen-activated protein kinase 14 | Mitogen-activated protein kinase 14 |  |
| LONP1 | Lon peptidase 1, mitochondrial | Lon protease homolog, mitochondrial |  |  |  |  |
| **Suppressors** |  |  |  |  |  |  |
| SLC7A11 | Solute carrier family 7 member 11 | Cystine/glutamate transporter | GPX4 | Glutathione peroxidase 4 | Phospholipid hydroperoxide glutathione peroxidase |  |
| AKR1C1 | Aldo-keto reductase family 1 member C1 | Aldo-keto reductase family 1 member C1 | AKR1C2 | Aldo-keto reductase family 1 member C2 | Aldo-keto reductase family 1 member C2 |  |
| AKR1C3 | Aldo-keto reductase family 1 member C3 | Aldo-keto reductase family 1 member C3 | RB1 | RB transcriptional corepressor 1 | Retinoblastoma-associated protein |  |
| HSPB1 | Heat shock protein family B (small) member 1 | Heat shock protein beta-1 | HSF1 | Heat shock transcription factor 1 | Heat shock factor protein 1 |  |
| GCLC | Glutamate-cysteine ligase catalytic subunit | Glutamate--cysteine ligase catalytic subunit | NFE2L2 | Nuclear factor, erythroid 2 like 2 | Nuclear factor erythroid 2-related factor 2 |  |
| SQSTM1 | Sequestosome 1 | Sequestosome-1 | LINC00336 | Long intergenic non-protein coding RNA 336 | Putative uncharacterized protein encoded by LINC00336 |  |
| HMOX1 | Heme oxygenase 1 | P09601 (HMOX1_HUMAN) | FTH1 | Ferritin heavy chain 1 | Ferritin heavy chain |  |
| MUC1 | Mucin 1, cell surface associated | Mucin-1 | SLC3A2 | Solute carrier family 3 member 2 | 4F2 cell-surface antigen heavy chain |  |
| MT1G | Metallothionein 1G | Metallothionein-1G | MTOR | Mechanistic target of rapamycin kinase | Serine/threonine-protein kinase mTOR |  |
| CISD1 | CDGSH iron sulfur domain 1 | CDGSH iron-sulfur domain-containing protein 1 | FANCD2 | FA complementation group D2 | Fanconi anemia group D2 protein |  |
| FTMT | Ferritin mitochondrial | Ferritin | HSPA5 | Heat shock protein family A (Hsp70) member 5 | Endoplasmic reticulum chaperone BiP |  |
| ATF4 | Activating transcription factor 4 | Cyclic AMP-dependent transcription factor ATF-4 | TP53 | Tumor protein p53 | Cellular tumor antigen p53 |  |
| HELLS | Helicase, lymphoid specific | Lymphoid-specific helicase | SCD | Stearoyl-CoA desaturase | Acyl-CoA desaturase |  |
| FADS2 | Fatty acid desaturase 2 | Acyl-CoA 6-desaturase | SRC | SRC proto-oncogene, non-receptor tyrosine kinase | Proto-oncogene tyrosine-protein kinase Src |  |
| STAT3 | Signal transducer and activator of transcription 3 | Signal transducer and activator of transcription 3 | PML | Promyelocytic leukemia | Protein PML |  |
| SLC40A1 | Solute carrier family 40 member 1 | Solute carrier family 40 member 1 | NFS1 | NFS1 cysteine desulfurase | Cysteine desulfurase |  |
| TP63 | Tumor protein p63 | Tumor protein 63 | CDKN1A | Cyclin dependent kinase inhibitor 1A | Cyclin-dependent kinase inhibitor 1 |  |
| MIR137 | microRNA 137 | _NA_ | ENPP2 | Ectonucleotide pyrophosphatase/phosphodiesterase 2 | Ectonucleotide pyrophosphatase/phosphodiesterase family member 2 | |
| VDAC2 | Voltage dependent anion channel 2 | Voltage-dependent anion-selective channel protein 2 | FH | Fumarate hydratase | Fumarate hydratase |  |
| CISD2 | CDGSH iron sulfur domain 2 | CDGSH iron-sulfur domain-containing protein 2 | MIR9-1 | microRNA 9-1 | _NA_ |  |
| MIR9-2 | microRNA 9-2 | _NA_ | MIR9-3 | microRNA 9-3 | _NA_ |  |
| CBS | Cystathionine beta-synthase | Cystathionine beta-synthase | ISCU | Iron-sulfur cluster assembly enzyme | Iron-sulfur cluster assembly enzyme ISCU |  |
| ACSL3 | Acyl-CoA synthetase long chain family member 3 | Long-chain-fatty-acid--CoA ligase 3 | OTUB1 | OTU deubiquitinase, ubiquitin aldehyde binding 1 | Ubiquitin thioesterase OTUB1 |  |
| CD44 | CD44 molecule (Indian blood group) | CD44 antigen | NQO1 | NAD(P)H quinone dehydrogenase 1 | NAD(P)H dehydrogenase [quinone] 1 |  |
| BRD4 | Bromodomain containing 4 | Bromodomain-containing protein 4 | TMBIM4 | Transmembrane BAX inhibitor motif containing 4 | Protein lifeguard 4 |  |
| MIR17 | microRNA 17 | _NA_ | SESN2 | Sestrin 2 | Sestrin-2 |  |
| NF2 | Neurofibromin 2 | Merlin | ARNTL | Aryl hydrocarbon receptor nuclear translocator like | Aryl hydrocarbon receptor nuclear translocator-like protein 1 |  |
| HIF1A | Hypoxia inducible factor 1 subunit alpha | Hypoxia-inducible factor 1-alpha | JUN | Jun proto-oncogene, AP-1 transcription factor subunit | Transcription factor AP-1 |  |
| CA9 | Carbonic anhydrase 9 | Carbonic anhydrase 9 | PRDX6 | Peroxiredoxin 6 | Peroxiredoxin-6 |  |
| PLIN2 | Perilipin 2 | Perilipin-2 | MIR212 | microRNA 212 | _NA_ |  |
| GCH1 | GTP cyclohydrolase 1 | GTP cyclohydrolase 1 | AIFM2 | Apoptosis inducing factor mitochondria associated 2 | Apoptosis-inducing factor 2 |  |
| LAMP2 | Lysosomal associated membrane protein 2 | Lysosome-associated membrane glycoprotein 2 | ZFP36 | ZFP36 ring finger protein | mRNA decay activator protein ZFP36 |  |
| PROM2 | Prominin 2 | Prominin-2 | CHMP5 | Charged multivesicular body protein 5 | Charged multivesicular body protein 5 |  |
| CHMP6 | Charged multivesicular body protein 6 | Charged multivesicular body protein 6 | CAV1 | Caveolin 1 | Caveolin-1 |  |
| Fer1HCH | Ferritin 1 Heavy Chain Homolog | Ferritin |  |  |  |  |
| **Markers** |  |  |  |  |  |  |
| PTGS2 | Prostaglandin-endoperoxide synthase 2 | P35354 (PGH2_HUMAN) | DUSP1 | Dual specificity phosphatase 1 | P28562 (DUS1_HUMAN) |  |
| NOS2 | Nitric oxide synthase 2 | P35228 (NOS2_HUMAN) | NCF2 | Neutrophil cytosolic factor 2 | P19878 (NCF2_HUMAN) |  |
| MT3 | Metallothionein 3 | P25713 (MT3_HUMAN) | UBC | Ubiquitin C | P0CG48 (UBC_HUMAN) |  |
| ALB | Albumin | P02768 (ALBU_HUMAN) | TXNRD1 | Thioredoxin reductase 1 | Q16881 (TRXR1_HUMAN) |  |
| SRXN1 | Sulfiredoxin 1 | Q9BYN0 (SRXN1_HUMAN) | GPX2 | Glutathione peroxidase 2 | P18283 (GPX2_HUMAN) |  |
| SNORA16A | Small nucleolar RNA, H/ACA box 16A | _NA_ | LOC390705 | _NA_ | _NA_ |  |
| SELENOS | Selenoprotein S | Q9BQE4 (SELS_HUMAN) | FTH1 | Ferritin heavy chain 1 | P02794 (FRIH_HUMAN) |  |
| CHAC1 | ChaC glutathione specific gamma-glutamylcyclotransferase 1 | Q9BUX1 (CHAC1_HUMAN) | SLC7A11 | Solute carrier family 7 member 11 | Q9UPY5 (XCT_HUMAN) |  |
| DDIT4 | DNA damage inducible transcript 4 | Q9NX09 (DDIT4_HUMAN) | RGS4 | Regulator of G protein signaling 4 | P49798 (RGS4_HUMAN) |  |
| ASNS | Asparagine synthetase (glutamine-hydrolyzing) | P08243 (ASNS_HUMAN) | TSC22D3 | TSC22 domain family member 3 | Q99576 (T22D3_HUMAN) |  |
| DDIT3 | DNA damage inducible transcript 3 | P0DPQ6 (DT3UO_HUMAN) | JDP2 | Jun dimerization protein 2 | Q8WYK2 (JDP2_HUMAN) |  |
| SESN2 | Sestrin 2 | P58004 (SESN2_HUMAN) | SLC1A4 | Solute carrier family 1 member 4 | P43007 (SATT_HUMAN) |  |
| PCK2 | Phosphoenolpyruvate carboxykinase 2, mitochondrial | Q16822 (PCKGM_HUMAN) | TXNIP | Thioredoxin interacting protein | Q9H3M7 (TXNIP_HUMAN) |  |
| VLDLR | Very low density lipoprotein receptor | P98155 (VLDLR_HUMAN) | GPT2 | Glutamic--pyruvic transaminase 2 | Q8TD30 (ALAT2_HUMAN) |  |
| PSAT1 | Phosphoserine aminotransferase 1 | Q9Y617 (SERC_HUMAN) | LURAP1L | Leucine rich adaptor protein 1 like | Q8IV03 (LUR1L_HUMAN) |  |
| HSD17B11 | Hydroxysteroid 17-beta dehydrogenase 11 | Q8NBQ5 (DHB11_HUMAN) | HERPUD1 | Homocysteine inducible ER protein with ubiquitin like domain 1 | Q15011 (HERP1_HUMAN) |  |
| XBP1 | X-box binding protein 1 | P17861 (XBP1_HUMAN) | ATF3 | Activating transcription factor 3 | P18847 (ATF3_HUMAN) |  |
| SLC3A2 | Solute carrier family 3 member 2 | P08195 (4F2_HUMAN) | CBS | Cystathionine beta-synthase | P35520 (CBS_HUMAN) |  |
| ATF4 | Activating transcription factor 4 | P18848 (ATF4_HUMAN) | ZNF419 | Zinc finger protein 419 | Q96HQ0 (ZN419_HUMAN) |  |
| KLHL24 | Kelch like family member 24 | Q6TFL4 (KLH24_HUMAN) | TRIB3 | Tribbles pseudokinase 3 | Q96RU7 (TRIB3_HUMAN) |  |
| ZFP69B | ZFP69 zinc finger protein B | Q9UJL9 (ZF69B_HUMAN) | ALOX5 | Arachidonate 5-lipoxygenase | P09917 (LOX5_HUMAN) |  |
| VEGFA | Vascular endothelial growth factor A | P15692 (VEGFA_HUMAN) | GDF15 | Growth differentiation factor 15 | Q99988 (GDF15_HUMAN) |  |
| TUBE1 | Tubulin epsilon 1 | Q9UJT0 (TBE_HUMAN) | ARRDC3 | Arrestin domain containing 3 | Q96B67 (ARRD3_HUMAN) |  |
| CEBPG | CCAAT enhancer binding protein gamma | P53567 (CEBPG_HUMAN) | IL6 | Interleukin 6 | P05231 (IL6_HUMAN) |  |
| SLC40A1 | Solute carrier family 40 member 1 | Q9NP59 (S40A1_HUMAN) | GABPB1 | GA binding protein transcription factor subunit beta 1 | Q06547 (GABP1_HUMAN) |  |
| OXSR1 | Oxidative stress responsive kinase 1 | O95747 (OXSR1_HUMAN) | EIF2S1 | Eukaryotic translation initiation factor 2 subunit 1 | P05198 (IF2A_HUMAN) |  |
| KIM-1 | Kidney injury molecule-1 | _NA_ | BNIP3 | BCL2 interacting protein 3 | Q12983 (BNIP3_HUMAN) |  |
| CXCL2 | C-X-C motif chemokine ligand 2 | P19875 (CXCL2_HUMAN) | RELA | RELA proto-oncogene, NF-kB subunit | Q04206 (TF65_HUMAN) |  |
| SLC7A5 | Solute carrier family 7 member 5 | Q01650 (LAT1_HUMAN) | AGPAT3 | 1-acylglycerol-3-phosphate O-acyltransferase 3 | Q9NRZ7 (PLCC_HUMAN) |  |
| SETD1B | SET domain containing 1B, histone lysine methyltransferase | Q9UPS6 (SET1B_HUMAN) | HMOX1 | Heme oxygenase 1 | P09601 (HMOX1_HUMAN) |  |
| TF | Transferrin | Serotransferrin | FTL | Ferritin light chain | P02792 (FRIL_HUMAN) |  |
| RPL8 | Ribosomal protein L8 | P62917 (RL8_HUMAN) | ATP5MC3 | ATP synthase membrane subunit c locus 3 | P48201 (AT5G3_HUMAN) |  |
| TFRC | Transferrin receptor | P02786 (TFR1_HUMAN) | MAFG | MAF bZIP transcription factor G | O15525 (MAFG_HUMAN) |  |
| IL33 | Interleukin 33 | O95760 (IL33_HUMAN) | ANGPTL7 | Angiopoietin like 7 | O43827 (ANGL7_HUMAN) |  |
| LOC284561 | _NA_ | _NA_ | GPX4 | Glutathione peroxidase 4 | P36969 (GPX4_HUMAN) |  |
| HAMP | Hepcidin antimicrobial peptide | P81172 (HEPC_HUMAN) | HSPB1 | Heat shock protein family B (small) member 1 | P04792 (HSPB1_HUMAN) |  |
| NFE2L2 | Nuclear factor, erythroid 2 like 2 | Q16236 (NF2L2_HUMAN) | STEAP3 | STEAP3 metalloreductase | Q658P3 (STEA3_HUMAN) |  |
| DRD5 | Dopamine receptor D5 | P21918 (DRD5_HUMAN) | DRD4 | Dopamine receptor D4 | P21917 (DRD4_HUMAN) |  |
| MAP3K5 | Mitogen-activated protein kinase kinase kinase 5 | Q99683 (M3K5_HUMAN) | MAPK14 | Mitogen-activated protein kinase 14 | Q16539 (MK14_HUMAN) |  |
| SLC2A1 | Solute carrier family 2 member 1 | P11166 (GTR1_HUMAN) | SLC2A3 | Solute carrier family 2 member 3 | P11169 (GTR3_HUMAN) |  |
| SLC2A6 | Solute carrier family 2 member 6 | Q9UGQ3 (GTR6_HUMAN) | SLC2A8 | Solute carrier family 2 member 8 | Q9NY64 (GTR8_HUMAN) |  |
| SLC2A12 | Solute carrier family 2 member 12 | Q8TD20 (GTR12_HUMAN) | GLUT13 | _NA_ | _NA_ |  |
| SLC2A14 | Solute carrier family 2 member 14 | Q8TDB8 (GTR14_HUMAN) | EIF2AK4 | Eukaryotic translation initiation factor 2 alpha kinase 4 | Q9P2K8 (E2AK4_HUMAN) |  |
| ATP6V1G2 | ATP6V1G2 | ATP6V1G2 | ALOX12 | Arachidonate 12-lipoxygenase, 12S type | P18054 (LOX12_HUMAN) |  |
| ALOX15 | Arachidonate 15-lipoxygenase | P16050 (LOX15_HUMAN) | ACSF2 | Acyl-CoA synthetase family member 2 | Q96CM8 (ACSF2_HUMAN) |  |
| IREB2 | Iron responsive element binding protein 2 | P48200 (IREB2_HUMAN) | HMGB1 | High mobility group box 1 | P09429 (HMGB1_HUMAN) |  |
| ELAVL1 | ELAV like RNA binding protein 1 | Q15717 (ELAV1_HUMAN) | TFAP2C | Transcription factor AP-2 gamma | Q92754 (AP2C_HUMAN) |  |
| NGB | Neuroglobin | Q9NPG2 (NGB_HUMAN) | MIR4715 | microRNA 4715 | _NA_ |  |
| NNMT | Nicotinamide N-methyltransferase | P40261 (NNMT_HUMAN) | PLIN4 | Perilipin 4 | Q96Q06 (PLIN4_HUMAN) |  |
| HIC1 | HIC ZBTB transcriptional repressor 1 | Q14526 (HIC1_HUMAN) | STMN1 | Stathmin 1 | P16949 (STMN1_HUMAN) |  |
| RRM2 | Ribonucleotide reductase regulatory subunit M2 | P31350 (RIR2_HUMAN) | CAPG | Capping actin protein, gelsolin like | P40121 (CAPG_HUMAN) |  |
| HNF4A | Hepatocyte nuclear factor 4 alpha | P41235 (HNF4A_HUMAN) | MIR30B | microRNA 30b | _NA_ |  |
| YWHAE | Tyrosine 3-monooxygenase/tryptophan 5-monooxygenase activation protein epsilon | P62258 (1433E_HUMAN) | SP1 | Sp1 transcription factor | P08047 (SP1_HUMAN) |  |
| AURKA | Aurora kinase A | O14965 (AURKA_HUMAN) | HBA1 | Hemoglobin subunit alpha 1 | P69905 (HBA_HUMAN) |  |
| RIPK1 | Receptor interacting serine/threonine kinase 1 | Q13546 (RIPK1_HUMAN) | PRDX1 | Peroxiredoxin 1 | Q06830 (PRDX1_HUMAN) |  |
| BLOC1S5-TXNDC5 | BLOC1S5-TXNDC5 readthrough (NMD candidate) | _NA_ |  |  |  |  |

**Supplemental Table 2.** Significant KEGG pathways for 6 differentially expressed ferroptosis-related genes.

| **KEGG term** | **Term description** | **Gene ID** | ***P* value** | ***p*. adjust** | **Q value** |
| --- | --- | --- | --- | --- | --- |
| hsa05167 | Kaposi sarcoma-associated herpesvirus infection | JUN/CDKN1A/ZFP36/CXCL2 | 4.51E-06 | 0.000406 | 0.000299 |
| hsa05166 | Human T-cell leukemia virus 1 infection | JUN/CDKN1A/ZFP36 | 0.000367 | 0.016508 | 0.012164 |
| hsa05211 | Renal cell carcinoma | JUN/CDKN1A | 0.001049 | 0.021047 | 0.015509 |
| hsa05120 | Epithelial cell signaling in Helicobacter pylori infection | JUN/CXCL2 | 0.001079 | 0.021047 | 0.015509 |
| hsa04012 | ErbB signaling pathway | JUN/CDKN1A | 0.001588 | 0.021047 | 0.015509 |
| hsa05210 | Colorectal cancer | JUN/CDKN1A | 0.001625 | 0.021047 | 0.015509 |
| hsa05323 | Rheumatoid arthritis | JUN/CXCL2 | 0.001898 | 0.021047 | 0.015509 |
| hsa04657 | IL-17 signaling pathway | JUN/CXCL2 | 0.001938 | 0.021047 | 0.015509 |
| hsa01522 | Endocrine resistance | JUN/CDKN1A | 0.002105 | 0.021047 | 0.015509 |
| hsa04668 | TNF signaling pathway | JUN/CXCL2 | 0.002740 | 0.024659 | 0.018170 |
| hsa05224 | Breast cancer | JUN/CDKN1A | 0.004675 | 0.035904 | 0.026456 |
| hsa04921 | Oxytocin signaling pathway | JUN/CDKN1A | 0.005121 | 0.035904 | 0.026456 |
| hsa04150 | mTOR signaling pathway | SLC7A5/DDIT4 | 0.005186 | 0.035904 | 0.026456 |
| hsa05161 | Hepatitis B | JUN/CDKN1A | 0.005654 | 0.036345 | 0.026780 |
| hsa04621 | NOD-like receptor signaling pathway | JUN/CXCL2 | 0.007018 | 0.042106 | 0.031026 |
| hsa05169 | Epstein-Barr virus infection | JUN/CDKN1A | 0.008685 | 0.046865 | 0.034532 |
| hsa05203 | Viral carcinogenesis | JUN/CDKN1A | 0.008852 | 0.046865 | 0.034532 |

**Supplemental Table 3.** Significant GO terms for 6 differentially expressed ferroptosis-related genes.

| **GO term** | **ID** | **Term description** | **Gene ID** | ***P* value** | ***p.* adjust** | **Q value** |
| --- | --- | --- | --- | --- | --- | --- |
| BP | GO:0051384 | response to glucocorticoid | CDKN1A/ZFP36/DDIT4 | 9.21E-06 | 0.003232 | 0.001327 |
| BP | GO:0031960 | response to corticosteroid | CDKN1A/ZFP36/DDIT4 | 1.26E-05 | 0.003232 | 0.001327 |
| BP | GO:0042594 | response to starvation | JUN/CDKN1A/ZFP36 | 2.06E-05 | 0.003232 | 0.001327 |
| BP | GO:0032897 | negative regulation of viral transcription | JUN/ZFP36 | 2.37E-05 | 0.003232 | 0.001327 |
| BP | GO:0042771 | intrinsic apoptotic signaling pathway in response to DNA damage by p53 class mediator | CDKN1A/DDIT4 | 8.47E-05 | 0.007825 | 0.003213 |
| BP | GO:0032496 | response to lipopolysaccharide | JUN/ZFP36/CXCL2 | 0.000105 | 0.007825 | 0.003213 |
| BP | GO:0048146 | positive regulation of fibroblast proliferation | JUN/CDKN1A | 0.000109 | 0.007825 | 0.003213 |
| BP | GO:0002237 | response to molecule of bacterial origin | JUN/ZFP36/CXCL2 | 0.000118 | 0.007825 | 0.003213 |
| BP | GO:0071385 | cellular response to glucocorticoid stimulus | ZFP36/DDIT4 | 0.000141 | 0.007825 | 0.003213 |
| BP | GO:0071384 | cellular response to corticosteroid stimulus | ZFP36/DDIT4 | 0.000156 | 0.007825 | 0.003213 |
| BP | GO:0048545 | response to steroid hormone | CDKN1A/ZFP36/DDIT4 | 0.000166 | 0.007825 | 0.003213 |
| BP | GO:0046782 | regulation of viral transcription | JUN/ZFP36 | 0.000172 | 0.007825 | 0.003213 |
| CC | GO:0005845 | mRNA cap binding complex | ZFP36 | 0.003647 | 0.034965 | 0.013384 |
| CC | GO:0034518 | RNA cap binding complex | ZFP36 | 0.004253 | 0.034965 | 0.013384 |
| CC | GO:0030014 | CCR4-NOT complex | ZFP36 | 0.005163 | 0.034965 | 0.013384 |
| CC | GO:0005719 | nuclear euchromatin | JUN | 0.009096 | 0.034965 | 0.013384 |
| CC | GO:1902555 | endoribonuclease complex | ZFP36 | 0.009398 | 0.034965 | 0.013384 |
| CC | GO:1905348 | endonuclease complex | ZFP36 | 0.009700 | 0.034965 | 0.013384 |
| CC | GO:0000791 | euchromatin | JUN | 0.011510 | 0.034965 | 0.013384 |
| CC | GO:0000307 | cyclin-dependent protein kinase holoenzyme complex | CDKN1A | 0.012715 | 0.034965 | 0.013384 |
| CC | GO:0010494 | cytoplasmic stress granule | ZFP36 | 0.019919 | 0.048556 | 0.018586 |
| CC | GO:0000932 | P-body | ZFP36 | 0.025294 | 0.048556 | 0.018586 |
| CC | GO:0017053 | transcriptional repressor complex | JUN | 0.025294 | 0.048556 | 0.018586 |
| CC | GO:1902554 | serine/threonine protein kinase complex | CDKN1A | 0.026485 | 0.048556 | 0.018586 |
| MF | GO:0071889 | 14-3-3 protein binding | ZFP36/DDIT4 | 3.87E-05 | 0.002130 | 0.000815 |
| MF | GO:0031625 | ubiquitin protein ligase binding | JUN/CDKN1A | 0.003843 | 0.039607 | 0.015161 |
| MF | GO:0004861 | cyclin-dependent protein serine/threonine kinase inhibitor activity | CDKN1A | 0.004062 | 0.039607 | 0.015161 |
| MF | GO:0044389 | ubiquitin-like protein ligase binding | JUN/CDKN1A | 0.004324 | 0.039607 | 0.015161 |
| MF | GO:0035497 | cAMP response element binding | JUN | 0.004738 | 0.039607 | 0.015161 |
| MF | GO:0071837 | HMG box domain binding | JUN | 0.005413 | 0.039607 | 0.015161 |
| MF | GO:0070412 | R-SMAD binding | JUN | 0.007774 | 0.039607 | 0.015161 |
| MF | GO:0019957 | C-C chemokine binding | ZFP36 | 0.008111 | 0.039607 | 0.015161 |
| MF | GO:0035925 | mRNA 3'-UTR AU-rich region binding | ZFP36 | 0.008784 | 0.039607 | 0.015161 |
| MF | GO:0017091 | AU-rich element binding | ZFP36 | 0.009457 | 0.039607 | 0.015161 |
| MF | GO:0030332 | cyclin binding | CDKN1A | 0.010130 | 0.039607 | 0.015161 |
| MF | GO:0015175 | neutral amino acid transmembrane transporter activity | SLC7A5 | 0.010466 | 0.039607 | 0.015161 |

**Supplemental Table 4.** Spearman correlation between immune cell subpopulations and *CDKN1A*.

| **Gene** | **Immune cells** | **Spearman coefficient** | ***P* value** | **Type** |
| --- | --- | --- | --- | --- |
| CDKN1A | T cells follicular helper | 0.286396 | 0.053655 | Positive |
| CDKN1A | B cells naive | 0.258066 | 0.083347 | Positive |
| CDKN1A | NK cells activated | 0.204866 | 0.172012 | Positive |
| CDKN1A | T cells CD8 | 0.180020 | 0.231244 | Positive |
| CDKN1A | Macrophages M1 | 0.068048 | 0.653183 | Positive |
| CDKN1A | T cells gamma delta | 0.054338 | 0.719849 | Positive |
| CDKN1A | T cells CD4 memory activated | -0.016821 | 0.911654 | Negative |
| CDKN1A | Macrophages M0 | -0.134076 | 0.374355 | Negative |
| CDKN1A | T cells regulatory (Tregs) | -0.154443 | 0.305446 | Negative |
| CDKN1A | Monocytes | -0.177597 | 0.237688 | Negative |
| CDKN1A | NK cells resting | -0.203152 | 0.175707 | Negative |
| CDKN1A | T cells CD4 naive | -0.251302 | 0.092054 | Negative |
| CDKN1A | B cells memory | -0.263420 | 0.076924 | Negative |
| CDKN1A | T cells CD4 memory resting | -0.292623 | 0.048441 | Negative |
| CDKN1A | Plasma cells | -0.503012 | 0.000367 | Negative |

**Supplemental Table 5.** Spearman correlation between immune cell subpopulations and *JUN*.

| **Gene** | **Immune cells** | **Spearman coefficient** | ***P* value** | **Type** |
| --- | --- | --- | --- | --- |
| JUN | T cells follicular helper | 0.249527 | 0.094451 | Positive |
| JUN | NK cells activated | 0.198512 | 0.185993 | Positive |
| JUN | Macrophages M1 | 0.194487 | 0.195258 | Positive |
| JUN | T cells gamma delta | 0.185353 | 0.217482 | Positive |
| JUN | T cells CD8 | 0.127770 | 0.397437 | Positive |
| JUN | B cells naive | 0.011738 | 0.938288 | Positive |
| JUN | T cells CD4 memory resting | -0.024780 | 0.870151 | Negative |
| JUN | T cells CD4 memory activated | -0.076518 | 0.613258 | Negative |
| JUN | T cells regulatory (Tregs) | -0.097059 | 0.521078 | Negative |
| JUN | Macrophages M0 | -0.122103 | 0.418871 | Negative |
| JUN | T cells CD4 naive | -0.244033 | 0.102180 | Negative |
| JUN | B cells memory | -0.254064 | 0.088417 | Negative |
| JUN | Monocytes | -0.285781 | 0.054194 | Negative |
| JUN | NK cells resting | -0.318156 | 0.031178 | Negative |
| JUN | Plasma cells | -0.330323 | 0.024958 | Negative |
